# Investigating the Effect of the Crosslinking Factor on the Properties of Hydrogel Materials Containing *Tilia platyphyllos* Hydrolate

**DOI:** 10.3390/molecules28207035

**Published:** 2023-10-11

**Authors:** Magdalena Kędzierska, Magdalena Bańkosz, Katarzyna Sala, Julia Dudzik, Piotr Potemski, Bożena Tyliszczak

**Affiliations:** 1 Department of Chemotherapy, Medical University of Lodz, Copernicus Memorial Hospital of Lodz, 90-549 Lodz, Poland; magdalena.kedzierska@umed.lodz.pl (M.K.); piotr.potemski@umed.lodz.pl (P.P.); 2 Department of Materials Engineering, Faculty of Materials Engineering and Physics, Cracow University of Technology, 37 Jana Pawła II Av., 31-864 Krakow, Poland; katarzyna.sala@student.pk.edu.pl (K.S.); julia.dudzik@student.pk.edu.pl (J.D.)

**Keywords:** hydrogel materials, *Tilia platyphyllos*, antioxidant properties, sorption capacity

## Abstract

The use of natural ingredients in recent years has been of great importance in many industries and medicine. In biomedical applications, hydrogel materials also play a significant role. In view of this, the aim of this study was to synthesize and characterize hydrogel materials enriched with broadleaf linden hydrolate. An important aspect was to carry out a series of syntheses with varying types and amounts of crosslinking agents so as to test the possibility of synthesizing materials with controlled properties. The obtained hydrogels were subjected to detailed physicochemical analysis. The results of the tests confirmed the relationship between the selected properties and the type of crosslinking agent used. A crosslinking agent with a lower molar mass (575 g/mol) results in a material with a compact and strongly crosslinked structure, which is characterized by high surface roughness. The use of a crosslinking agent with a molecular weight of 700 g/mol resulted in a material with a looser-packed polymer network capable of absorbing larger amounts of liquids. The work also proved that regardless of the type of crosslinking agent used, the addition of linden hydrolate provides antioxidant properties, which is particularly important in view of the target biomedical application of such materials.

## 1. Introduction

Modern medicine is developing at an extremely fast pace, which is due to the constant search for new and better solutions to provide patients with effective medical care and improve their quality of life [1,2]. Materials that have long been gaining the attention of researchers due to their unique properties are hydrogels. Three-dimensional polymer networks are materials that have made significant progress in recent years and are increasingly being used by researchers to create advanced polymer matrices. Their unique properties make them widely used in various fields, such as medicine, cosmetology and materials engineering [3,4,5]. The three-dimensional networks are characterized by a high capacity to bind and store water, making them flexible and able to take any shape [6,7]. Hydrogels are also biocompatible, meaning that they are safe for living organisms and can be used in a variety of medical applications, such as wound dressings, artificial tissues or drug carriers and many others [8,9,10]. Their widespread use is causing researchers to increasingly look for ways to modify them to obtain materials with specific properties that could be used to solve a variety of medical problems [11]. Modification of hydrogels can include changing their structure, adding different components to them or implementing a different production method [12]. Such procedures make it possible to obtain hydrogels with a certain porosity, better permeability to oxygen and nutrients or greater resistance to external agents [13,14]. All this makes hydrogels an increasingly important topic of scientific research, and their development has great potential for applications in both the medical and cosmetic fields [15]. We can modify hydrogels by adding various types of plant extracts with different properties, depending on what role the finished material will play. An example of this modification is wound dressings in the form of hydrogel, which contain natural plant extracts, that is, aqueous or alcoholic plant isolates obtained by extraction [16,17]. Dressings impregnated with natural plant extracts are now widely used to impart antimicrobial properties to modern wound dressings. In particular, the electrospinning method has proven suitable for producing wound dressings containing plant extracts and essential oils. Encapsulation of these natural compounds in nanofiber mats enhances their antimicrobial activity by transforming the plant material into a relatively stable structure and improves the release profile due to a high surface-to-volume ratio compared to using the plant material in its pure form [18].

Polyethylene glycol diacrylate (PEGDA) hydrogel crosslinking is a chemical process that involves the formation of permanent chemical bonds between polymer particles to form a three-dimensional hydrogel structure. The process is widely used in the pharmaceutical, medical, cosmetic and other industries due to the ability of hydrogels to retain and release active ingredients and their ability to retain water [19]. A mixture of selected monomers, in which PEGDA is also present, is mixed with a suitable reaction initiator, e.g., in the case of UV light polymerization, a photoinitiator. Under UV light of the appropriate wavelength for the initiator, photons absorbed by the photoinitiator promote its cleavage, which leads to the formation of free radicals [20,21]. These extremely reactive molecules then react with the vinyl bonds of the monomers used and also with the PEGDA chain, which leads to the formation of chemical bonds between the polymer chains. This reaction depends on many factors, but mainly on the components selected in the process and their concentrations [22,23]. This process makes it possible to obtain hydrogels with controlled mechanical properties and an appropriate degree of degradation. Hydrogels obtained in this way have the ability to absorb and retain water and other substances, which makes them useful in many uses, such as in the production of dressings, controlled-release drug capsules, contact lenses and many other medicinal and cosmetic products [24].

**Figure 1:**
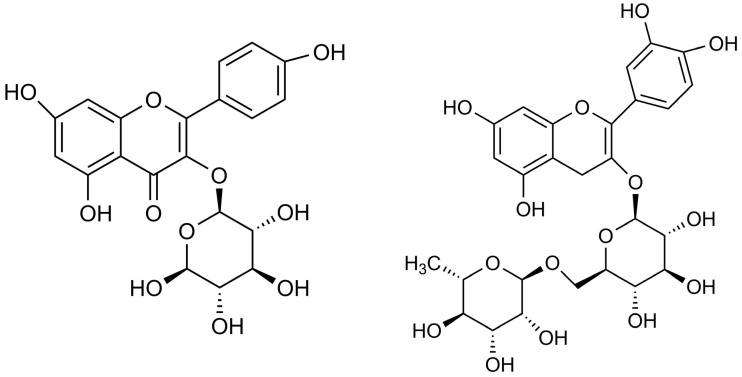
Structural formula of astragalin (**left**) and rutin (**right**) found in linden extract.

The use of broad-leaved linden may be of interest. The flowers of this plant contain 3–10% mucilaginous polysaccharides, mainly arabinogalactans and uronic acids; 2% tannins (procyanidins); 1% flavonoids, mainly quercetin glycosides (rutin, hyperoside, quercitrin, isoquercitrin) and kaempferol (astragalin); and phenolic acids (caffeic acid, p-coumaric acid, chlorogenic acid). The structural formula of astragalin and rutin found in the linden extract is presented in Figure 1. The essential oil extracted from the flowers mainly contains terpene tricosane and oxidized isocyclocyclic monoterpenes and hotrienol [19,20,21]. The pharmacological activity of linden inflorescence is attributed to flavonoids, mainly kemferol and quercetin. These compounds have low molecular weight and high bioactivity with antioxidant, anti-inflammatory, antiviral and anticancer activities [22,23]. The flowers have antispasmodic, expectorant, emollient, mildly hypotensive, laxative, and sedative properties. The sedative effect is attributed to the presence of farnesol in the essential oil. The flower infusion is used for colds, coughs, and runny noses, as well as indigestion, high blood pressure, anxiety, hysteria, and nervous vomiting. The flower extract has also been shown to contribute to the disappearance of lymphoma cells, inhibit mitogen-induced proliferation of normal lymphocytes and have hepatoprotective properties. Volatile compounds have antibacterial properties [24,25]. The cosmetic and dermatological importance of the flowers is mainly due to their content of mucilaginous polysaccharides, which act on the skin as a moisturizing and anti-inflammatory. Polysaccharides absorb large amounts of water due to their high hydroxyl content. These hydrocolloids can be applied to the skin in the form of a film, thus releasing water into the stratum corneum and protecting the skin tissue. Linden blossom extract is used in masks for moisturizing, soothing and against redness, and is suitable for dry and sensitive skin [26,27]. For example, Mastuda et al. in their study confirmed the hepaprotective effect of linden extract [28]. Subsequently, Kruk et al. demonstrated the anti-inflammatory effect of this sub-stance. In biological analysis, it was confirmed that substances included in the linden extract lead to increased production of SCFA (short-chain fatty acids) and thus inhibit the production of pro-inflammatory cytokines [29]. Special attention is paid to the study of the antioxidant properties of this plant. Also in the present study, these properties were analyzed. However, literature data confirm the excellent antioxidant activity of the plant used. Poljsak et al. showed that an increase in the amount of tocopherol increases the antioxidant properties [27]. These properties were also confirmed by Cittan et al. in [30] turn, Cárdenas-Rodríguez et al. proved the anticonvulsant effect of preparations based on linden plant extract [26].

The aim of this work was to produce hydrogels with the addition of linden hydrolate for biomedical and cosmetic applications. The main goal of this work was to obtain hydrogel materials with high application potential using an environmentally friendly and economical process of photopolymerization in the UV field. The main process that can change the properties of the material is changing the degree of crosslinking. An increase in the value of the degree of crosslinking will increase the strength of the hydrogel matrix and, in the case of biodegradable materials, allow longer degradation times. The disadvantage of this solution is an increase in brittleness. In order to obtain a hydrogel with sufficient flexibility, it is necessary to find the right value for the degree of crosslinking. Therefore, in this work, hydrogel materials with different amounts of the poly(ethylene glycol) diacrylate (PEGDA) cross-linking agent were synthesized, which additionally differed in molecular weight (575 g/mol and 700 g/mol, respectively). The scope of the experimental work included the appropriate preparation of samples for testing, analysis of the swelling capacity in selected fluids and incubation testing. In addition, the scope included FT-IR spectroscopy analysis, antioxidant activity analysis, microscopic observations of hydrogel surfaces and determination of the roughness profile. All analyses were aimed at determining the significance of the type and amount of crosslinking agent and linden hydrolate on the physicochemical properties of the modified hydrogels. A special aspect of the work and a research novelty is the combination of linden plant hydrolate with a hydrogel matrix. The uses of this plant described so far mainly concerned its use in the form of extracts and infusions as ingredients of ointments or creams. The combination of this plant hydrolate, rich in natural ingredients, with a hydrogel material may prove to be an innovative solution in biomedical applications, with particular emphasis on dressing materials.

## 2. Results and Discussion

### 2.1. Analysis of Sorption Capacity

**Figure 2:**
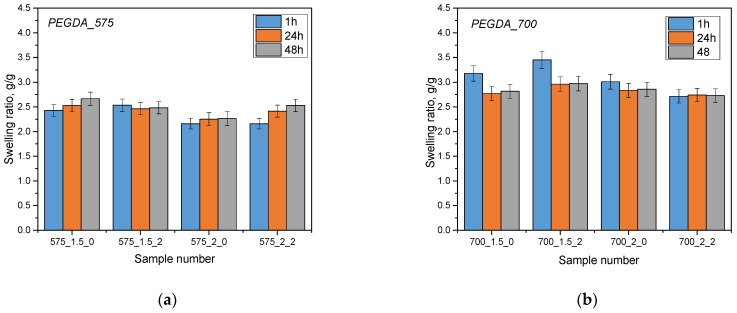

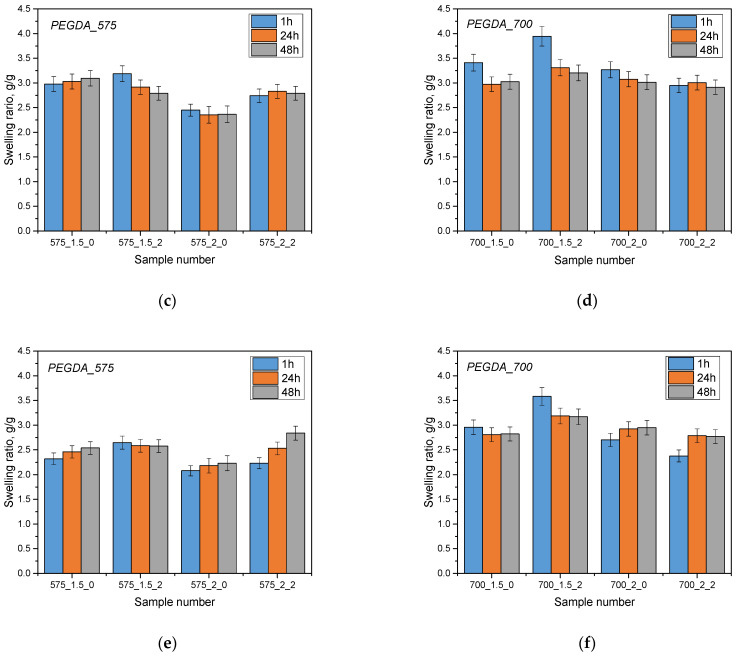
Results of sorption capacity analysis in Ringer’s solution (**a**,**b**), distilled water (**c**,**d**) and PBS liquid (**e**,**f**) (*n*—number of repetitions, *n* = 3).

The aim of the analysis was to investigate the sorption capacity of hydrogel materials by analyzing their swelling. The swelling process is a key parameter in assessing the absorption capacity of different liquids. Samples of hydrogel materials were tested in three different liquids: Ringer’s solution, phosphate buffer (PBS) and distilled water. The results of this analysis are presented below (Figure 2).

The hydrogel materials tested showed sorption capabilities for the various fluids that were used for the test. Additionally, it has been noted that the use of a 575 g/mol molecular crosslinking agent resulted in lower swelling coefficient values compared to a 700 g/mol crosslinking agent. This is due to differences in the structure of hydrogel materials obtained using these different crosslinking agents. A lower molecular weight crosslinking agent (575 g/mol) leads to shorter crosslinkages between the polymer chains, which increases the crosslinking density of the polymer matrix. As a result, this reduces fluid ingress and swelling of materials. In contrast, a higher molecular weight crosslinking agent (700 g/mol) results in longer chains, resulting in lower crosslinking density and easier filling of the space by the absorbed liquid, resulting in higher swelling coefficient values. In addition, the addition of linden hydrolate did not significantly affect the swelling coefficient values, which may indicate that the sorption properties of the developed materials were preserved in the presence of this modifier. On the other hand, the results of statistical analysis show significant differences when different crosslinking agents and analyses were used in different incubation fluids (Table 1).

Swelling kinetics showed that the greatest changes in swell rate occurred within the first hour and then stabilized after 24 and 48 h. The highest values of the swelling coefficient occurred in samples immersed in distilled water. This is probably due to the fact that the distilled water does not contain ions, which may influence additional interactions and crosslinking in the hydrogels, thus limiting fluid penetration. For distilled water, where ions are lacking, the sorption process is not limited, resulting in the highest swelling coefficient values for this liquid. The opposite is true for Ringer’s liquids and PBS buffer. Polymer chains forming a three-dimensional hydrogel network can be connected by chemical bonds, but also their structure can be supported by electrostatic interactions, hydrogen bonds or as a result of hydrophobic interactions. A number of different interactions affect the structure of the hydrogel network and its crosslinking density. In the case of liquids containing monovalent or multivalent ions, additional crosslinks or physical interactions between the chains and components of the selected liquid can occur. Substitutions of the extracted ions and their interactions with the polymer network can lead to an increase in its density by which the sorption activity of the hydrogel becomes limited. This is important for the design of carrier-like materials. Such materials should exhibit an appropriate degree of swelling, which simultaneously ensures the loosening of the polymer network and the release of components deposited inside the material, including active substances such as drugs or compounds with anti-inflammatory and antioxidant activity. The developed materials can be successfully used in various applications. Due to the possibility of changing the amount and type of crosslinking agent, it is possible to design a material with closely correlated and desired properties.

### 2.2. Results of the Incubation Study

**Figure 3:**
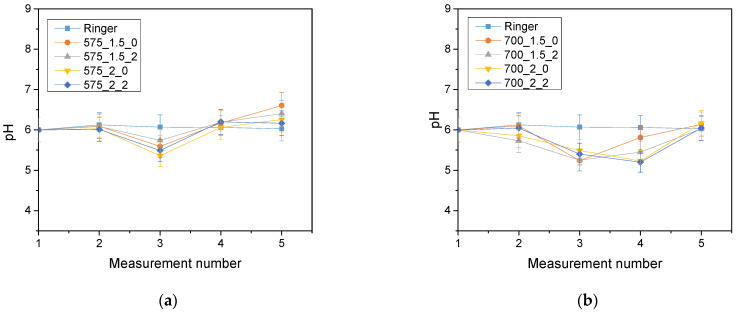
Results of incubation analysis in Ringer’s solution for samples with crosslinking agent 575 g/mol (**a**) and for samples with crosslinking agent 700 g/mol (**b**).

**Figure 4:**
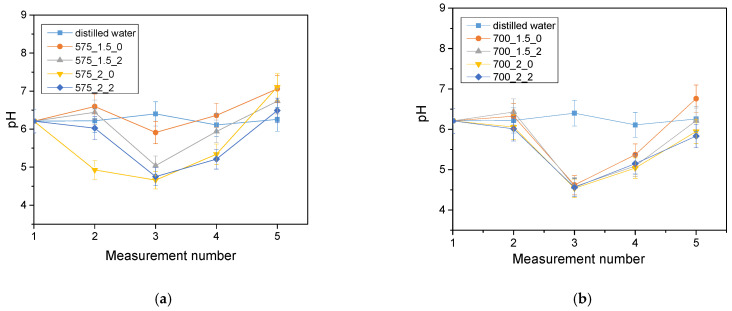
Results of incubation analysis in distilled water for samples with crosslinking agent 575 g/mol (**a**) and for samples with crosslinking agent 700 g/mol (**b**).

**Figure 5:**
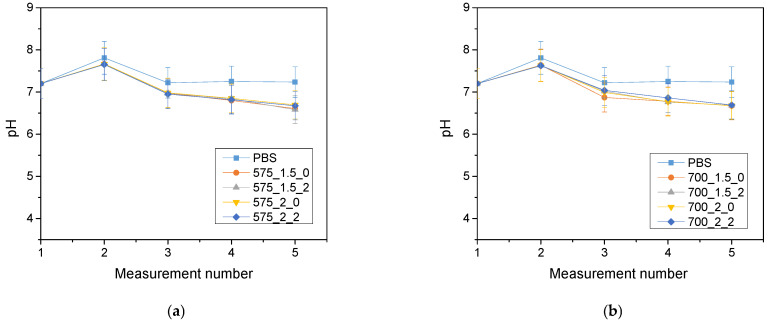
Results of incubation analysis in PBS for samples with crosslinking agent 575 g/mol (**a**) and for samples with crosslinking agent 700 g/mol (**b**) (n—number of repetitions, *n* = 3).

The analysis of the incubation study was performed to determine the change in pH and closely related temperature, illustrating the effect of the hydrogel on the properties of the fluid in which it was found. The results of the analysis performed for Ringer’s solution, distilled water and PBS are presented below (Figure 3, Figure 4 and Figure 5).

The largest pH changes were observed in samples immersed in distilled water, while the most stable pH values were maintained in samples immersed in PBS fluid. PBS liquid is known as a buffer solution, which has minimal pH change even after the addition of strong acids or bases or dilution with water. In contrast, distilled water and Ringer’s fluid showed initial drops in pH, after which pH values increased over time and reached values higher than the initial ones. The pH jumps were most noticeable after 72 h of incubation. It is noteworthy, however, that these changes were not drastic, typically amounting to only one pH unit. This suggests that the hydrogel materials tested were not completely degraded. Such pH changes may be due to the process of swelling of the hydrogel material, which absorbs the incubation liquid and, at the same time, may release substances retained in its matrix, such as polymeric components or linden hydrolate. Linden hydrolate, which is a modifier of the materials used, is a complex substance containing compounds of both basic and acidic nature. The release of these compounds and their interaction with the incubation fluid can cause changes in pH values, as observed in the analysis. Considering the antioxidant and anti-inflammatory properties of the extract from this plant, the release of active substances is a desired effect.

### 2.3. Results of Antioxidant Activity Analysis

**Figure 6:**
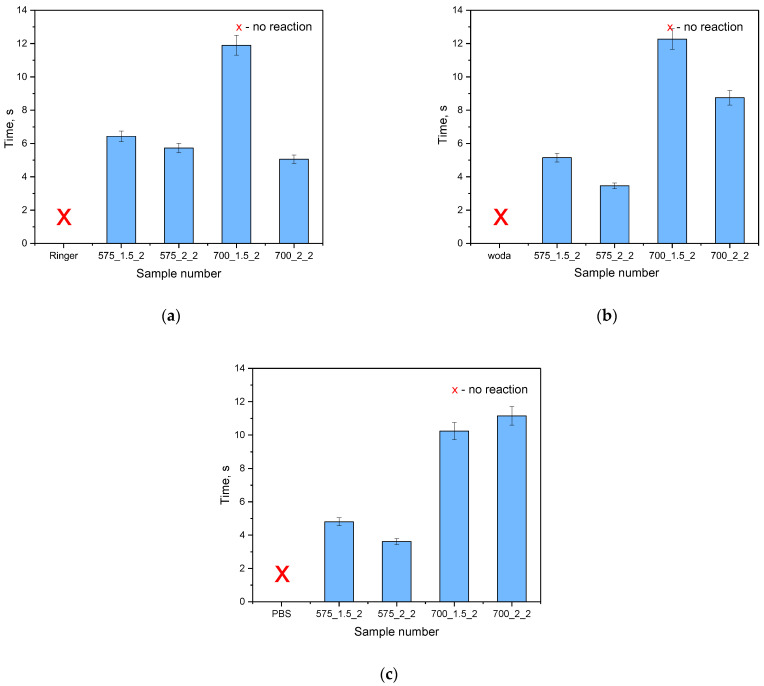
Results of antioxidant activity analysis of samples incubated in Ringer’s liquid (**a**) distilled water (**b**) and PBS (**c**) (n—number of repetitions, n = 3).

**Figure 7:**
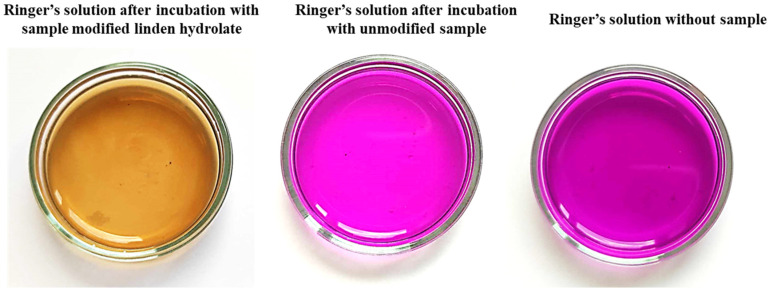
Change in color during testing of antioxidant properties.

To confirm the results of the incubation study on the potential release of active substances, an analysis of antioxidant activity was conducted. Incubation fluids that previously contained hydrogel materials were tested. An incubation fluid in which there was no hydrogel material and a fluid in which materials not modified with linden hydrolate were placed were tested as reference samples. The results of the survey are presented in Figure 6 and Figure 7.

Table 2 presents the results of the statistical analysis for the antioxidant capacity test.

Antioxidants are compounds that, even in small amounts, protect the body from the effects of free radicals. In addition, antioxidants slow down oxidation reactions in the body, take part in the body’s defense against bacteria or viruses, and counteract the proliferation of free radicals, i.e., molecules unpaired by an electron, which, striving for “pairing”, take these electrons from healthy cells. Antioxidant activity is characteristic of many plant extracts, including linden. In view of this, during the analysis, the possibility of reacting compounds released from the prepared hydrogel materials with potassium permanganate (VII) was checked. This compound takes on a dark purple color characteristic of Mn^VII+^ manganate ions. When these ions interact with the incubation fluids, the compounds released from the hydrogel react with potassium permanganate (KMnO_4_), leading to a reduction of Mn^VII+^ ions and a change in the color of the fluid, suggesting the antioxidant activity of the tested samples as presented in Figure 7.

The antioxidant capacity was checked for both samples modified with linden hydrolate and those not modified with this additive. During the test, the time it took for the KMnO_4_ solution to decolorize to a brownish-yellow color indicative of the reduction of Mn^VII+^ ions to Mn^II+^ was measured. For all the materials tested that contained linden hydrolate, these changes were noted to occur within a few seconds or so. It is interesting to note that for hydrogel materials containing only polymeric components, no color change was observed. Thus, the possibility of interaction with potassium permanganate by possible compounds from the polymer network was ruled out. The incubation fluid itself, in which no sample was placed, was also used as a reference sample. Again, the color change did not occur. Thus, it can be concluded that the above incubation results and the analysis of antioxidant properties are consistent. There is a release of active substances from the hydrogel material, which exhibit antioxidant activity in just a few seconds.

### 2.4. Results of Infrared Spectroscopy Analysis

**Figure 8:**
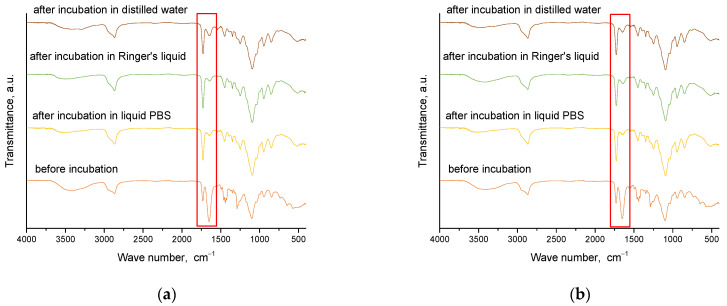

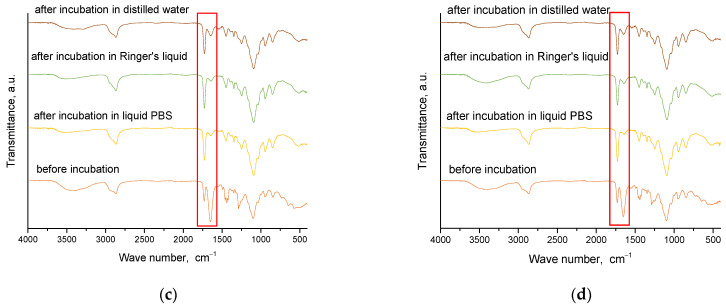
FT-IR spectroscopic analysis results for sample series 575: 1.5_0 (**a**); 1.5_2 (**b**); 2_0 (**c**); 2_2 (**d**) (the red frame indicates absorption bands of varying intensity).

**Figure 9:**
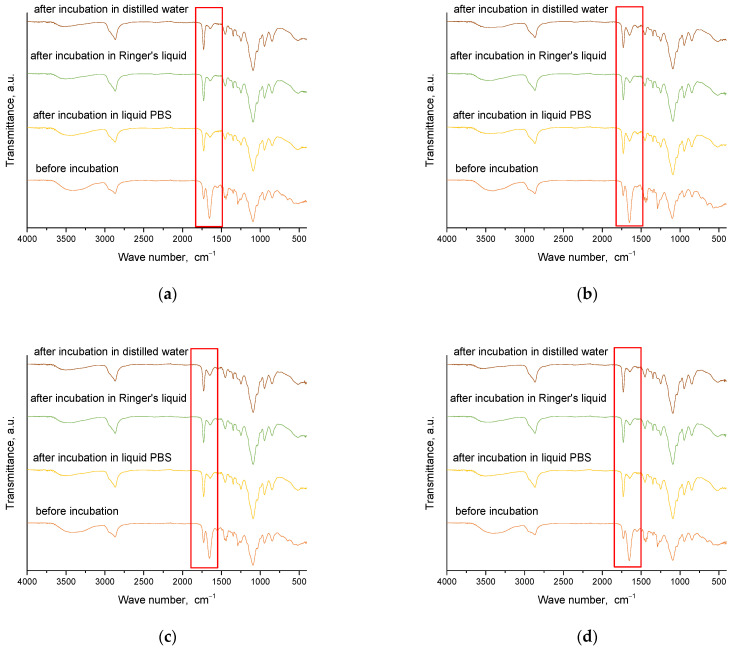
FT-IR spectroscopic analysis results for sample series 700: 1.5_0 (**a**); 1.5_2 (**b**); 2_0 (**c**); 2_2 (**d**) (the red frame indicates absorption bands of varying intensity).

Spectroscopic analysis was carried out for materials obtained immediately after synthesis and for those that were incubated. This analysis aims to determine the effect of incubation on the structure of these materials. The results of this study are presented in Figure 8 and Figure 9.

**Figure 10:**
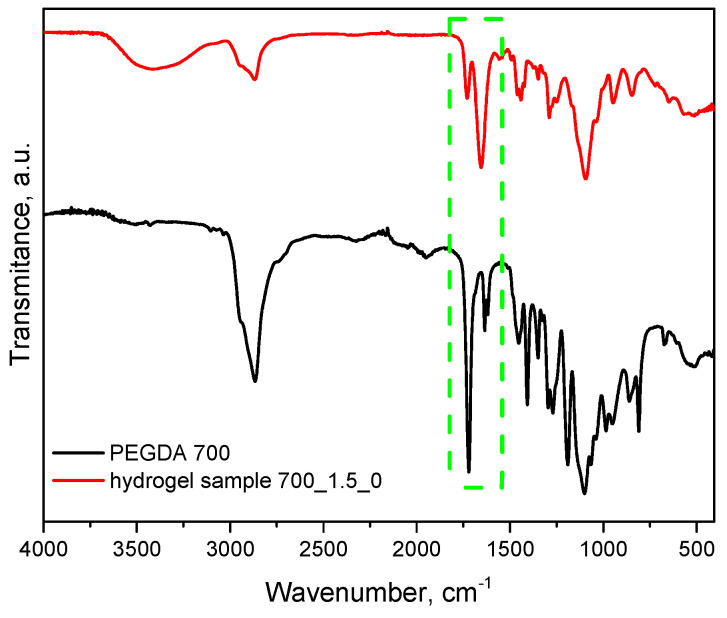
FT-IR spectra of uncrosslinked PEGDA 700 g/mol crosslinking agent (black line) and an example hydrogel material (red line) (the green box indicates the bands characteristic of cross-linked PEGDA).

Figure 10 additionally presents a comparison of FT-IR spectra of an example sample of hydrogel material with a crosslinking agent before the crosslinking process. As can be clearly seen, the absorption band characteristic of the C=C double bond occurring in the uncrosslinked PEGDA agent in the hydrogel significantly reduces its intensity (1725 cm^−1^). The reduction in the intensity of this band indicates that the crosslinking process is occurring correctly, which, in turn, confirms the choice of an appropriate synthesis method and parameters.

Table 3 shows the identified absorption bands together with the corresponding type of vibration and characteristic bonds.

From the analysis, characteristic absorption bands were found for PVP and gelatin polymers, as well as the crosslinking agent PEGDA. An absorption band associated with the stretching vibration of the -OH groups (in the range of 3646–3291 cm^−1^) was identified, and its maximum was at 3432 cm^−1^. Another characteristic band associated with the stretching vibration of the C=O group was detected at 1653 cm^−1^. A bending band with a characteristic CH_2_ bond was seen in the range of 1429–1411 cm^−1^, with a maximum at 1423 cm^−1^. The results of the analysis showed that the absorption bands characteristic of the hydrogel components were very similar for all the materials studied. After incubating the samples in different liquids, no significant changes were observed in the absorption bands. Nevertheless, a slight reduction in the intensity of selected bands was noted, especially the band characteristic of the vibration of the -OH group and the C=O bond. These characteristic bonds are present both in the components of the polymer matrix and in the linden hydrolate, which was used as a modifier. Based on the results obtained, it can be concluded that there was release of the active ingredient, the linden hydrolate. The results are, therefore, consistent with those presented above.

### 2.5. Optical Microscope Observations

Observation using an optical microscope was performed to visualize and compare the surfaces of the hydrogel materials. Images from microscopic observations are presented in Table 4.

More undulations can be observed on the surface of samples containing a crosslinking agent with a molecular weight of 575 g/mol compared to samples with a 700 g/mol agent. This is due to the difference in the molecular weight of the crosslinking agent used. The higher number of visible corrugations on the surface of the 575-series samples is due to the fact that the crosslinks are shorter than those of the 700 g/mol factor samples. Shorter bonds result in denser crosslinking of the hydrogel material. The addition of linden hydrolate affected the structure of the individual samples. The surfaces of the hydrogels with the additive showed fewer corrugations compared to the samples without the additive. Microscopic images confirm that hydrogels with a higher amount of crosslinking agent (2 mL) had a more undulating structure than samples with a lower amount (1.5 mL) of this agent. This indicates that the amount of crosslinking agent used also has a significant effect on the hydrogel structure. Moreover, the samples in the 700 series appear to be less homogeneous, and the individual bubbles on their surface are due to intensive mixing during sample preparation.

### 2.6. Digital Microscope Observations and Roughness Profile Analysis

**Figure 11:**
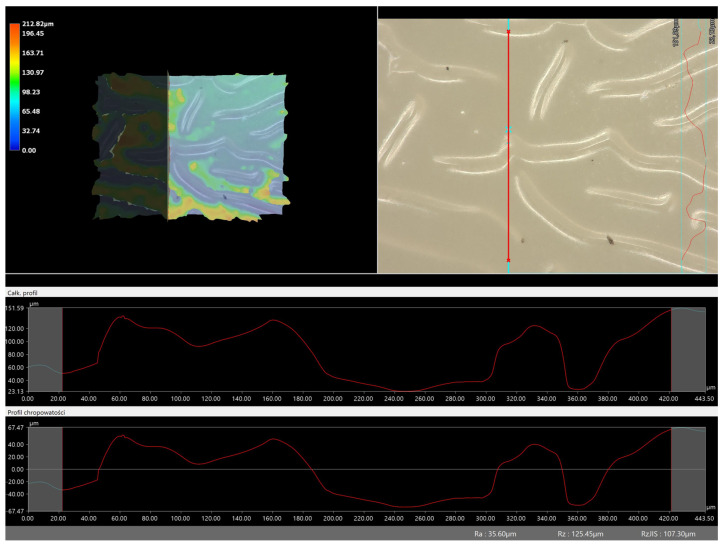
Roughness profile image for sample containing 575 g/mol crosslinking agent (sample 575_2.0._2) (the red line indicates the roughness profile).

Next, the microscopic observations were supplemented with the use of a digital microscope, enabling the evaluation of the surface roughness profile of the obtained hydrogel materials. The results of the analysis are presented in Table 5 and Table 6, as well as Figure 10 and Figure 11.

**Figure 12:**
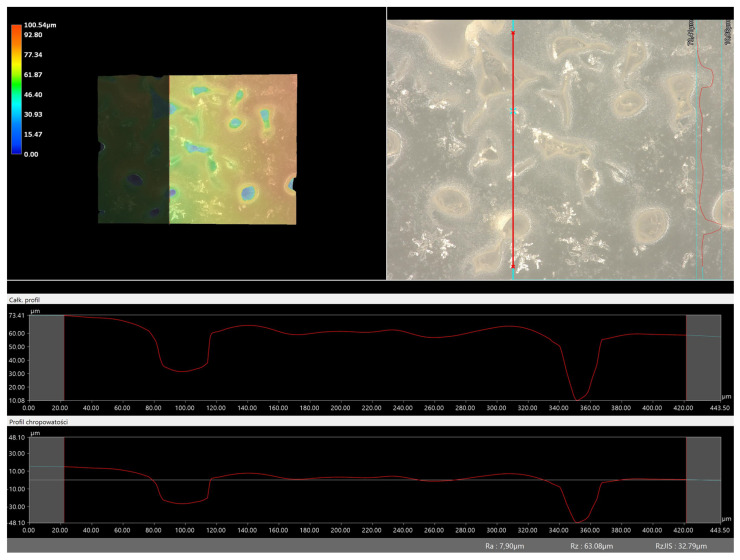
Roughness profile image for sample containing 700 g/mol crosslinking agent (sample 700_2.0._2) (the red line indicates the roughness profile).

The digital microscope images presented in Table 5 fully correspond to the microscopic images presented in Table 4. With the amount of crosslinking agent, we observed a change in the degree of crosslinking of the hydrogel material. In addition, greater surface corrugation was observed for all materials obtained with a crosslinking agent with a molecular weight of 575 g/mol. The analysis of microscopic observations was also supplemented by determining the surface roughness profile of these materials. Figure 11 and Figure 12 present an example of how the roughness profile was determined for a sample containing a crosslinking agent of 575 g/mol (Figure 11) and 700 g/mol (Figure 12), respectively.

Analysis of surface roughness measurements was carried out for all the materials obtained, both those modified with linden hydrolate and those unmodified. The values determining the basic roughness parameter Ra (absolute average relative to the base length) are presented in Table 6.

Analysis of the surface roughness profile clearly confirmed consistent results with microscopic observations. As the amount of crosslinking agent increased in both cases (575 and 700), the surface corrugation and roughness increased. Larger values of the roughness parameter Ra were recorded for all materials obtained with a crosslinking agent with a molecular weight of 575 g/mol than for 700 g/mol. As already indicated by previous analyses, the use of this type of crosslinking agent results in the formation of a polymer network with a more compact, dense and more strongly crosslinked structure. This also results in surface changes in this material and greater roughness. For example, for the sample containing crosslinking agent 575, the roughness parameter is 35.60 µm, while for the same type of material obtained with 700 g/mol PEGDA, the value is much lower (7.90 µm). Based on the results obtained, it can be concluded that the surface properties are closely related to the composition of the obtained materials and can be easily adjusted to the desired effects.

### 2.7. Analysis of Mechanical Properties

**Figure 13:**
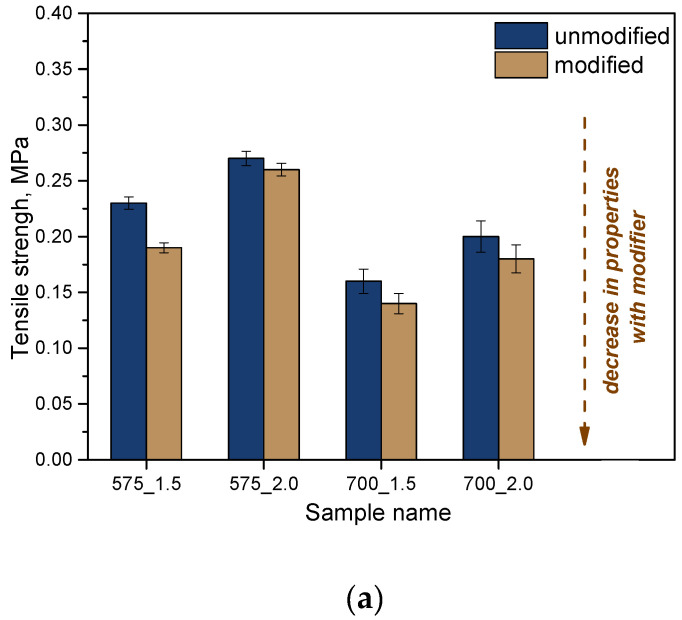

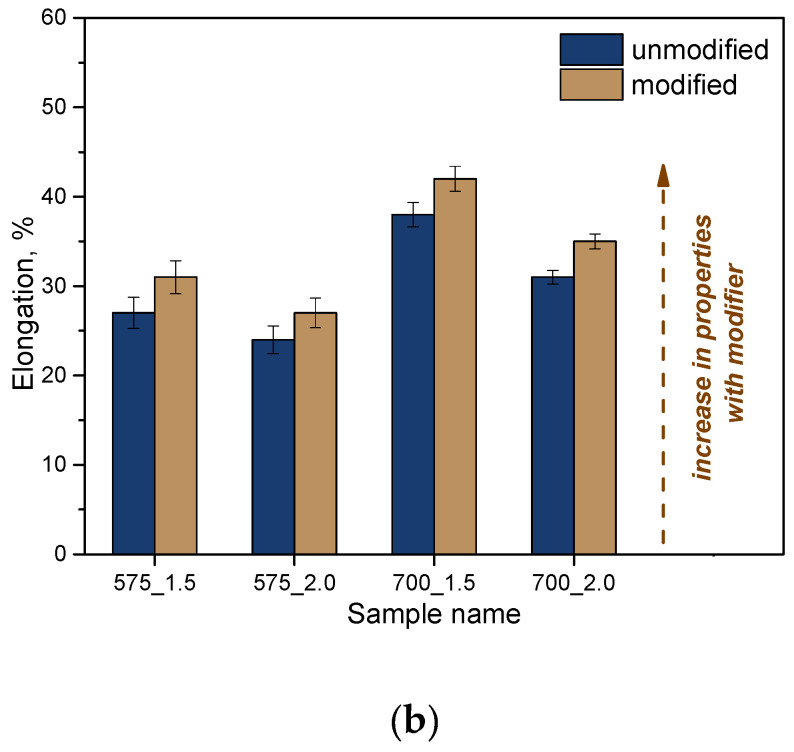
Results of mechanical property analysis: tensile strength (**a**); percent elongation (**b**) (n—number of repetitions, n = 3).

Then, the analysis of strength properties was carried out. Considering the potential application of the developed materials as dressing materials, it is extremely important to determine their tensile strength and percentage elongation. The materials should be both strong and flexible enough to facilitate their application at the wound site. The results of the strength analysis are presented in Figure 13.

Based on the analysis, it was shown that the molecular weight of the crosslinking agent affects the strength properties of the materials obtained. In the case of the crosslinking agent PEGDA with a molecular weight of 575 g/mol, higher tensile strength was shown at the same time with lower elasticity than for samples obtained with PEGDA 700 g/mol. Increasing the weight of the crosslinking agent leads to a change in the internal structure of the crosslinked polymeric materials. The higher molecular weight leads to a more loosely packed network of polymer chains, which are longer, which translates into greater flexibility of these materials. If an additional modifier is used while keeping the same proportions of crosslinking agent and photoinitiator, there is a deterioration in the strength of these materials. On the other hand, their flexibility is increased. Considering the intended use in dressing applications, the increase in flexibility is a desirable phenomenon and another advantage of the obtained systems.

## 3. Materials and Methods

### 3.1. Materials

Poly(vinylpyrrolidone) (powder, average mol wt. 58,000), gelatin (from porcine skin, gel strength 300, Type A), poly(ethylene glycol) diacrylate (PEGDA, crosslinker, average molecular weight Mn = 700 g/mol and Mn = 575 g/mol), 2-hydroxy-2-methylpropiophenone (photoinitiator, d = 1.077 g/mL, 97%) were bought from Merck (Darmstadt, Germany). In turn, linden hydrolate (99.38% Tilia platyphyllos (linden) flower water; product is certified by the ECOCERT organization for its ecological purity) was purchased from BiochemiaUrody (Raciborz, Poland). Hydrolate obtained by steam distillation of the flowers of the broad-leaved linden tree of the genus Tilia Platyphyllos Sp from organic cultivation. The substance in the form of a light yellow, transparent liquid, with a fine sediment.

### 3.2. Synthesis of Hydrogel Materials

The synthesis process was initiated by preparing the appropriate solutions—a 15% PVP solution and a 2% gelatine solution. Appropriate amounts of these solutions were then taken and mixed in a specific order. Subsequently, a crosslinking agent was added to the polymer mixture and, in the final step, a photoinitiator—2-hydroxy-2-methylpropiophenone—was included. The mixture was then transferred to a Petri dish and placed under a UV lamp (specifically an EMITA VP-60 lamp with a power of 180 W and a wavelength (λ) of 320 nm). The sample was irradiated for 120 s. After the irradiation process, the material was dried and prepared for further studies. An analogous synthesis process was carried out for samples containing linden extract, in which case the extract was added before the crosslinking agent and photoinitiator.

**Figure 14:**
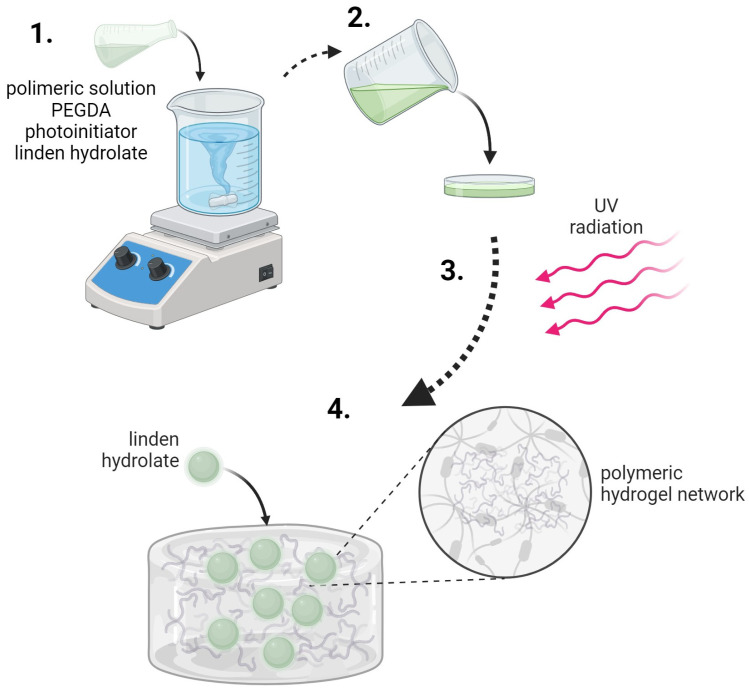
The scheme for obtaining hydrogel materials (steps in the synthesis of hydrogel materials: (**1**) preparation of the reaction mixture; (**2**) selection of the polymerization form; (**3**) polymerization under UV light; (**4**) the obtained hydrogel material).

Hydrogel materials were obtained by photopolymerization using a PEGDA crosslinking agent of various molecular weights. The diagram of the crosslinking process is presented in Figure 14. The polymer chains of polyvinylpyrrolidone and gelatin interact with each other, and the crosslinking agent causes additional crosslinking of the internal hydrogel structure. A variable amount of the crosslinking agent changes the internal structure of such a material and, thus, its properties. The analysis of these changes was the main subject of this research.

The composition of each hydrogel is shown in Table 7.

The sample markings in the Table above correspond to the markings in the results presented in Section 3. The name of the sample refers to the molecular weight of the crosslinking agent (575 or 700) and then to the amount of the agent used (1.5 or 2.0) and the presence or absence of an additive in the form of linden hydrolate (0 or 2). For example, the sample marked 575_1.5_2 contains 1.5 mL of PEGDA crosslinking agent with a molecular weight of 575 g/mol and 2 mL of modifying agent.

The scheme for obtaining hydrogel materials is presented in Figure 14.

### 3.3. Sorption Capacity Analysis

The main objective of this study was to determine the sorptive capacity of the hydrogel materials tested in the context of swelling. For therapeutic dressings, it is crucial that the dressing material has adequate swelling capacity, meaning that it can absorb exudate from the wound. To carry out the swelling analysis, small discs with a diameter of 1 cm (for each series of materials) were used. The discs were carefully dried and weighed. Then, the samples were placed in 50 mL of different liquids: Ringer’s solution, PBS solution and distilled water. After 1 h, the discs were removed, drained of excess liquid and reweighed. Analogous weight measurements were carried out after 24 and 48 h. For each sample, 3 repetitions were performed to minimize measurement errors. Finally, the swelling coefficient results obtained from the repeated tests were averaged to obtain a representative value of the sorption capacity of the hydrogel matrices. On the basis of the analysis, the degree of swelling was determined by determining the sorption coefficient α (Equation (1)).

(1) $$\alpha =\frac{m_{t}-m_{0}}{m_{0}}$$

where:

α—swelling ratio, g/g;
m_t_—mass of swollen sample after time “t”, g;
m_0_—mass of dry sample (before the study), g.

### 3.4. Incubation Studies in Simulated Body Fluids

The aim of the incubation studies is to show the interaction between the hydrogel matrix and solutions corresponding to human physiological fluids. Changes in pH values may indicate leaching of uncrosslinked agents or cause degradation of samples in fluids, among other things. To carry out the test, discs with a diameter of 1 cm were sterilely placed in containers to which 50 mL of three selected fluids were added: Ringer’s solution, PBS and distilled water. The samples were incubated at 37 °C in an incubator. The pH values were measured using a CX-701 ELMETRON multifunctional device for 10 days, with measurements every other day.

### 3.5. Analysis of Antioxidant Properties

**Figure 15:**
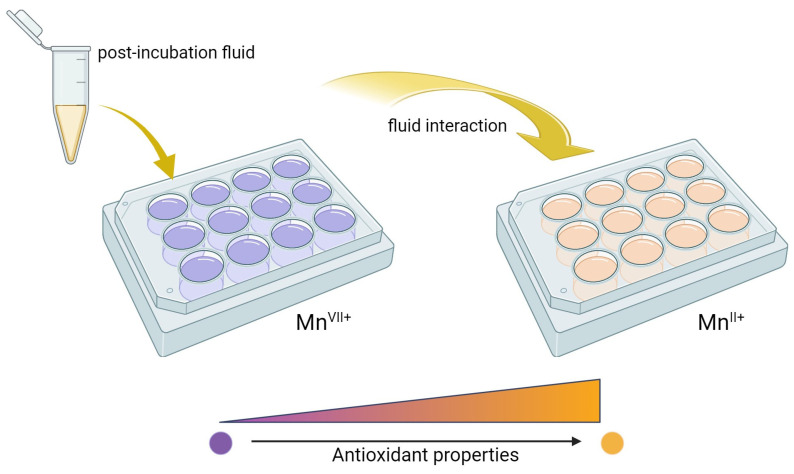
Scheme of analysis of antioxidant properties.

To complement the incubation studies, an analysis of antioxidant capacity was conducted. The chosen method aims to determine or exclude the antioxidant effect of substances released during the incubation of hydrogel materials. All hydrogel materials, both containing modifier and unmodified, were tested. An incubation fluid in which no sample was present was used as a reference sample. To carry out the test, the interaction of the test sample with potassium permanganate, which is a universal reagent that oxidizes a number of chemical compounds, was selected, and the effect of this oxidation is a change in the color of individual samples. Post-incubation fluids were separated from the hydrogel material after a 10-day incubation. A few drops of 0.1% potassium permanganate were added to 3 mL of each fluid, and the reaction was observed while measuring the time it took for the test sample to change color. If a given sample did not discolor within 1 h, the material was considered to have no antioxidant properties. In each case, a threefold repetition was carried out. The scheme of this analysis is presented in Figure 15.

### 3.6. Characterization of the Chemical Structure of Hydrogel Materials via Fourier Transform Infrared (FT-IR) Spectroscopy

Spectroscopic analysis was carried out for all samples, both those modified with linden hydrolate and those that remained in unmodified form. Included in the study was a sample that was collected and dried immediately after synthesis, as well as samples that had been incubated in their respective liquids for a period of ten days. For the analysis, a Thermo Scientific Nicolet iS5 spectrometer was used, which was equipped with an ATR (Attenuated Total Reflectance) stand. Absorption spectra were recorded at room temperature in the wavelength range 4000 to 600 cm^−1^.

### 3.7. Observation using an Optical Microscope

In order to accurately assess the surface characteristics and structure of the synthesized hydrogel matrices, microscopic observations were made using a Delta Optical Genetic Pro optical microscope. Images were taken at ×40 magnification. The main objective of these analyses was to visualize and compare the changes in surface structure as a result of different crosslinking agents and the introduction of linden hydrolate into the hydrogel matrices.

### 3.8. Observation Using a Digital Microscope and Determination of Surface Roughness

Subsequently, microscopic observations were made using a Keyence 4K VKX-7000 digital microscope (Keyence, Osaka, Japan) that captured high-resolution images. The device is equipped with a REMAX CEO and a 4K CMOS image sensor, providing high accuracy and magnification. The analysis carried out was intended to complement the observations made with the optical microscope as well as to determine the surface roughness profile of the materials obtained.

### 3.9. Analysis of Mechanical Properties

A strength assessment was conducted to analyze the mechanical characteristics of the hydrogels. The mechanical investigations adhered to the guidelines outlined in standards ISO 527-2 type 5A and ISO 37 type 2 [31,32]. As a result of the analysis, the tensile strength and percentage elongation of the tested materials were determined.

### 3.10. Statistical Analysis

In the case of analyses for which multiple repetitions were performed, statistical ANOVA (two-way analysis of variance with repetitions) was performed. This analysis made it possible to determine statistical significance at the assumed confidence level α = 0.05.

## 4. Conclusions

Based on the results obtained, it was concluded that the tested samples exhibited sorptive and antioxidant properties, which will enable their further use as wound dressings or for cosmetic purposes as masks or hydrogel pads. The advantage of the tested materials is that, depending on their further application, we can modify them accordingly to obtain the desired properties. It has been proven that the type and amount of crosslinking agent used has a decisive influence on the structure of the material. Hydrogels containing a crosslinking agent PEGDA of 575 g/mol were characterized by a more undulating material surface than those with a crosslinking agent PEGDA of 700 g/mol. It was also found that as the molecular weight of the crosslinking agent increased, the swelling coefficients increased. The modifier in the form of linden hydrolate, through the release of individual active substances, can influence the pH of a given solution. However, the analysis of antioxidant properties confirmed that the samples containing linden hydrolate show the desired properties, which proves the release of active substances from the hydrogel material. Also, the results of the spectroscopic analysis confirmed the possibility of releasing the active additive. The obtained materials show great application potential and can be the subject of further detailed research.

## Figures and Tables

**Table 1:** Statistical analysis of obtained data based on the two-way analysis of variance (ANOVA) with repetitions (for sorption ability).

Independent Variable	Sum of Squares	Mean Square	f-Value	*p*-Value
Type of incubation fluid	1.0225	0.5112	3681.16	1.8567 × 10^−17^
Type of crosslinking agent	1.7174	1.7174	12,365.44	1.8741 × 10^−19^
Interaction	0.0754	0.0377	271.72	1.0168 × 10^−10^

At the 0.05 level, the population means of “type of incubation fluid” are significantly different. At the 0.05 level, the population means of “composition of sample” are significantly different. At the 0.05 level, the interaction between both factors is significant.

**Table 2:** Statistical analysis of obtained data based on the two-way analysis of variance (ANOVA) with repetitions (for antioxidant property).

Independent Variable	Sum of Squares	Mean Square	f-Value	*p*-Value
Type of incubation fluid	8.5297	4.2648	3239.13	3.9949 × 10^−17^
Type of crosslinking agent	160.622	160.622	121,992.12	2.0425 × 10^−25^
Interaction	3.1109	1.5554	1181.38	1.6647 × 10^−14^

At the 0.05 level, the population means of “type of incubation fluid” are significantly different. At the 0.05 level, the population means of “composition of sample” are significantly different. At the 0.05 level, the interaction between both factors is significant.

**Table 3:** Summary of characteristic bonds and vibrations for the obtained absorption band values.

Wave Number [cm^−1^]	Vibration Type	Characteristic Binding
3432 (3646–3291)	tensile	O-H
2868 (2911–2835)	tensile	CH_2_
1731 (1756–1708)	tensile	C=O
1653 (1705–1585)	tensile	C=O
1423 (1429–1411)	deformation (bending)	CH_2_
1289 (1311–1280)	tensile	C-N

**Table 4:** Images of sample surfaces under an optical microscope for the 575 and 700 series.

Sample Number	Series 575	Series 700
1.5_0	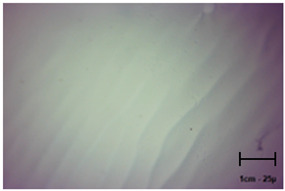	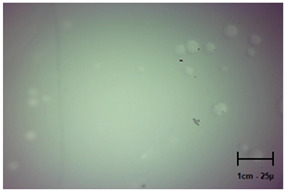
1.5_2	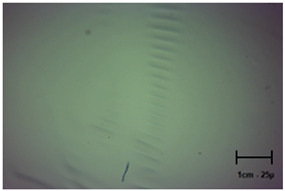	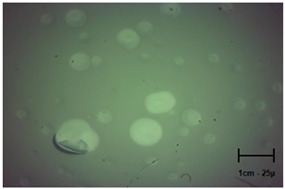
2.0_0	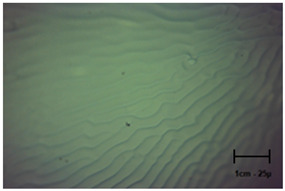	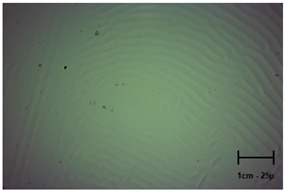
2.0_2	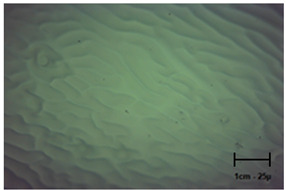	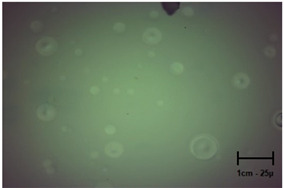

**Table 5:** Images of sample surfaces under a digital microscope for the 575 and 700 series.

Sample Number	Series 575	Series 700
1.5_0	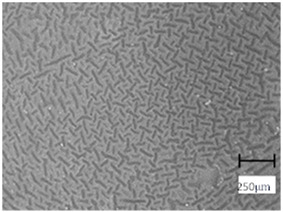	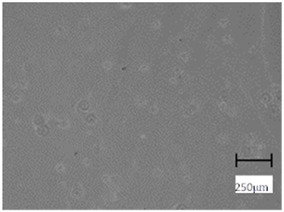
1.5_2	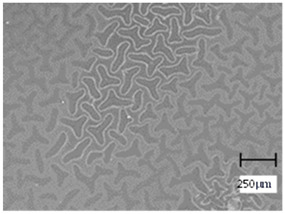	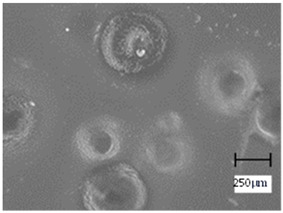
2.0_0	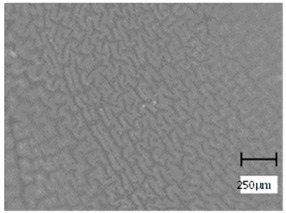	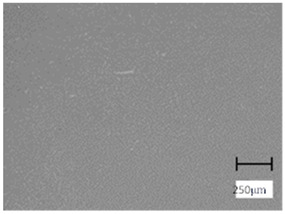
2.0_2	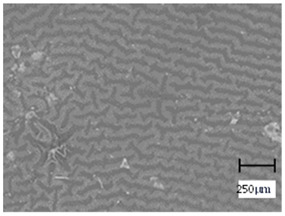	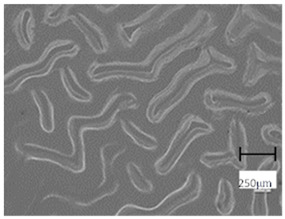

**Table 6:** Results of surface roughness analysis of hydrogel materials.

Sample Number	Ra, µm Series 575	Ra, µm Series 700
1.5_0	17.52	5.70
1.5_2	11.61	3.63
2.0_0	35.60	7.90
2.0_2	20.38	6.35

**Table 7:** Composition of hydrogel materials.

Base Solution *	PEGDA 575 g/mol, [mL]	PEGDA 700 g/mol, [mL]	Photoinitiator, [µL]	Linden Hydrolate, [mL]	Sample Name
10	1.5	-		-	575_1.5_0
	1.5	-		2.0	575_1.5_2
	2.0	-		-	575_2.0_0
	2.0	-		2.0	575_2.0_2
	-	1.5		-	700_1.5_0
	-	1.5	50	2.0	700_1.5_2
	-	2.0		-	700_2.0_0
	-	2.0		2.0	700_2.0_2

* Base solution: 5 mL 15% PVP and 5 mL 2% gelatin.

## Data Availability

Data sharing is not applicable for this article.

## References

[1] Peppas N.A., Hilt J.Z., Khademhosseini A., Langer R. (2006). Hydrogels in Biology and Medicine: From Molecular Principles to Bionanotechnology. Adv. Mater..

[2] Dong Y.C., Bouché M., Uman S., Burdick J.A., Cormode D.P. (2021). Detecting and Monitoring Hydrogels with Medical Imaging. ACS Biomater. Sci. Eng..

[3] Adorinni S., Rozhin P., Marchesan S. (2021). Smart Hydrogels Meet Carbon Nanomaterials for New Frontiers in Medicine. Biomedicines.

[4] Yang K., Han Q., Chen B., Zheng Y., Zhang K., Li Q., Wang J. (2018). Antimicrobial Hydrogels: Promising Materials for Medical Application. Int. J. Nanomed..

[5] Tayler I.M., Stowers R.S. (2021). Engineering Hydrogels for Personalized Disease Modeling and Regenerative Medicine. Acta Biomater..

[6] Bashir S., Hina M., Iqbal J., Rajpar A.H., Mujtaba M.A., Alghamdi N.A., Wageh S., Ramesh K., Ramesh S. (2020). Fundamental Concepts of Hydrogels: Synthesis, Properties, and Their Applications. Polymers.

[7] Ho T.-C., Chang C.-C., Chan H.-P., Chung T.-W., Shu C.-W., Chuang K.-P., Duh T.-H., Yang M.-H., Tyan Y.-C. (2022). Hydrogels: Properties and Applications in Biomedicine. Molecules.

[8] Chamkouri H. (2021). A Review of Hydrogels, Their Properties and Applications in Medicine. Am. J. Biomed. Sci. Res..

[9] Cao H., Duan L., Zhang Y., Cao J., Zhang K. (2021). Current Hydrogel Advances in Physicochemical and Biological Response-Driven Biomedical Application Diversity. Signal Transduct. Target. Ther..

[10] Chyzy A., Plonska-Brzezinska M.E. (2020). Hydrogel Properties and Their Impact on Regenerative Medicine and Tissue Engineering. Molecules.

[11] Huang G., Tang Z., Peng S., Zhang P., Sun T., Wei W., Zeng L., Guo H., Guo H., Meng G. (2022). Modification of Hydrophobic Hydrogels into a Strongly Adhesive and Tough Hydrogel by Electrostatic Interaction. Macromolecules.

[12] Du H., Shi S., Liu W., Teng H., Piao M. (2020). Processing and Modification of Hydrogel and Its Application in Emerging Contaminant Adsorption and in Catalyst Immobilization: A Review. Environ. Sci. Pollut. Res..

[13] Sánchez-Cid P., Jiménez-Rosado M., Romero A., Pérez-Puyana V. (2022). Novel Trends in Hydrogel Development for Biomedical Applications: A Review. Polymers.

[14] Zhang K., Yan W., Simic R., Benetti E.M., Spencer N.D. (2020). Versatile Surface Modification of Hydrogels by Surface-Initiated, Cu0-Mediated Controlled Radical Polymerization. ACS Appl. Mater. Interfaces.

[15] Cui L., Yao Y., Yim E.K.F. (2021). The Effects of Surface Topography Modification on Hydrogel Properties. APL Bioeng..

[16] Micale N., Citarella A., Molonia M.S., Speciale A., Cimino F., Saija A., Cristani M. (2020). Hydrogels for the Delivery of Plant-Derived (Poly)Phenols. Molecules.

[17] Zahid M., Lodhi M., Afzal A., Rehan Z.A., Mehmood M., Javed T., Shabbir R., Siuta D., Althobaiti F., Dessok E.S. (2021). Development of Hydrogels with the Incorporation of *Raphanus Sativus* L. Seed Extract in Sodium Alginate for Wound-Healing Application. Gels.

[18] Bianchera A., Catanzano O., Boateng J., Elviri L. (2020). The Place of Biomaterials in Wound Healing. Therapeutic Dressings and Wound Healing Applications.

[19] Neirynck J., Mirtcheva S., Sioen G., Lust N. (2000). Impact of *Tilia platyphyllos* Scop., *Fraxinus excelsior* L., *Acer pseudoplatanus* L., *Quercus robur* L. and *Fagus sylvatica* L. on Earthworm Biomass and Physico-Chemical Properties of a Loamy Topsoil. For. Ecol. Manag..

[20] Phuekvilai P., Wolff K. (2013). Characterization of Microsatellite Loci in *Tilia platyphyllos* (Malvaceae) and Cross-Amplification in Related Species. Appl. Plant Sci..

[21] Saygi K.O., Cacan E. (2021). Antioxidant and Cytotoxic Activities of Silver Nanoparticles Synthesized Using Tilia Cordata Flowers Extract. Mater. Today Commun..

[22] Szűcs Z., Cziáky Z., Kiss-Szikszai A., Sinka L., Vasas G., Gonda S. (2019). Comparative Metabolomics of *Tilia platyphyllos* Scop. Bracts during Phenological Development. Phytochemistry.

[23] Ferreira T., Nascimento-Gonçalves E., Macedo S., Borges I., Gama A., Gil da Costa R.M., Neuparth M.J., Lanzarin G., Venâncio C., Félix L. (2021). Toxicological and Anti-Tumor Effects of a Linden Extract (*Tilia platyphyllos* Scop.) in a HPV16-Transgenic Mouse Model. Food Funct..

[24] Siger A., Antkowiak W., Dwiecki K., Rokosik E., Rudzińska M. (2021). Nutlets of *Tilia cordata* Mill. and *Tilia platyphyllos* Scop.—Source of Bioactive Compounds. Food Chem..

[25] Symma N., Bütergerds M., Sendker J., Petereit F., Hake A., Düfer M., Hensel A. (2021). Novel Piperidine and 3,4-Dihydro-2H-Pyrrole Alkaloids from *Tilia platyphyllos* and *Tilia cordata* Flowers. Planta Med..

[26] Cárdenas-Rodríguez N., González-Trujano M.E., Aguirre-Hernández E., Ruíz-García M., Sampieri A., Coballase-Urrutia E., Carmona-Aparicio L. (2014). Anticonvulsant and Antioxidant Effects of *Tilia americana* var. Mexicana and Flavonoids Constituents in the Pentylenetetrazole-Induced Seizures. Oxid. Med. Cell. Longev..

[27] Poljšak N., Kočevar Glavač N. (2021). *Tilia* sp. Seed Oil—Composition, Antioxidant Activity and Potential Use. Appl. Sci..

[28] Matsuda H., Ninomiya K., Shimoda H., Yoshikawa M. (2002). Hepatoprotective Principles from the Flowers of *Tilia argentea* (Linden): Structure Requirements of Tiliroside and Mechanisms of Action. Bioorganic Med. Chem..

[29] Kruk A., Granica S., Popowski D., Malinowska N., Piwowarski J.P. (2022). Tiliae Flos Metabolites and Their Beneficial Influence on Human Gut Microbiota Biodiversity Ex Vivo. J. Ethnopharmacol..

[30] Cittan M., Altuntaş E., Çelik A. (2018). Evaluation of Antioxidant Capacities and Phenolic Profiles in *Tilia cordata* Fruit Extracts: A Comparative Study to Determine the Efficiency of Traditional Hot Water Infusion Method. Ind. Crops Prod..

[31] (2017). Rubber, Vulcanized or Thermoplastic—Determination of Tensile Stress-Strain Properties.

[32] (2012). Determination of Mechanical Properties in Static Tension—Part 2: Test Conditions for Plastics Intended for Various Molding Techniques.

